# Exploration of the relationship between apoptosis related characteristic genes and the prognosis of HCC

**DOI:** 10.7150/jca.114359

**Published:** 2025-07-28

**Authors:** Qiyao Zhang, Zhen Cao, Hongtao Cao, Hao Wu, Shangcheng Yan, Yuqian Kan, Xinwei Cui, Yingchun Feng, Ziwen Liu

**Affiliations:** Department of General Surgery, Peking Union Medical College Hospital, Chinese Academy of Medical Sciences and Peking Union Medical College, Beijing 100730, P. R. China.

**Keywords:** hepatocellular carcinoma, anoikis, prognosis, molecular subtype

## Abstract

The acquisition of resistance to anoikis is a critical driver of metastasis in various tumor types. However, the combined role of anoikis apoptosis in the progression and prognosis of hepatocellular carcinoma (HCC) remains largely unexplored. This study integrates known anoikis genes with single-cell datasets to identify differentially expressed Anoikis (DE-Anoikis) through unsupervised clustering, enabling the classification of samples from The Cancer Genome Atlas (TCGA). A prognostic risk model was constructed using univariate Cox proportional hazards regression and validated with external datasets from the International Cancer Genome Consortium (ICGC) and the Gene Expression Omnibus (GEO). The results revealed significant prognostic differences among DE-Anoikis-based HCC molecular subtypes, with functional enrichment analyses highlighting metabolic reprogramming differences. Furthermore, the anoikis-related prognostic model demonstrated robust predictive accuracy across multiple validation datasets. Two potential therapeutic drugs exhibited sensitivity in low-risk patients, offering novel insights into HCC treatment. Overall, this study identifies a unique subgroup of apoptosis-associated HCC and a prognostic model, providing further biological insights into the molecular mechanisms and therapeutic strategies for HCC.

## Introduction

Hepatocellular carcinoma (HCC) is the most common primary liver cancer, accounting for 90% of hepatic cancers 1, 2. The high prevalence of hepatitis B virus (HBV) infection in East Asia and Africa has led to a higher incidence of HCC in these regions compared to other parts of the world 3, 4. Treatment options for HCC depend on clinical and pathological staging and include liver transplantation, liver resection (LR), transarterial chemoembolization (TACE), transarterial radioembolization (TARE), chemotherapy, and radiation therapy 5, 6. In recent years, the combination of targeted therapies and immunotherapy has significantly improved the prognosis for patients with advanced HCC, marking a milestone in oncology treatment 7-9. However, the insidious onset and rapid progression of HCC make early diagnosis, metastasis prevention, and treatment resistance challenging. Understanding the mechanisms of HCC metastasis is crucial for identifying potential therapeutic targets.

Metastasis is a key event in HCC progression and a major factor contributing to the poor prognosis of patients. HCC metastasis involves the separation of cancer cells from the primary tumor and their spread to other organs, primarily via the circulatory system 10, 11. Normally, the detachment of cells from the extracellular matrix triggers a form of programmed cell death called apoptosis. However, tumor cells acquire resistance to this "anoikis" (detachment-induced apoptosis), enabling them to survive in an anchorage-independent manner 12-14. This resistance extends their survival in the circulatory system and facilitates their colonization at secondary sites 15. Acquisition of anoikis resistance is a critical factor in HCC metastasis. Several proteins have been shown to play key roles in anoikis resistance. For example, the 14-3-3ζ protein inhibits the intrinsic apoptotic pathway by attenuating Bad and p53 activity, thereby conferring anoikis resistance to cancer cells. Additionally, 14-3-3σ promotes anoikis resistance in HCC by regulating the EGFR-ERK1/2 pathway 16.

In this study, we screened key regulators of anoikis using single-cell sequencing data from HCC samples. By clustering the HCC samples based on these genes, we identified two distinct anoikis-related HCC molecular subtypes. Functional enrichment analysis provided further insights into potential biological mechanisms. We used univariate Cox regression, least absolute shrinkage and selection operator (LASSO) regression, and multivariate Cox regression analyses to identify prognostic genes and construct prognostic risk models. These models were then evaluated with respect to the immune microenvironment and chemotherapeutic drug sensitivity to explore potential sensitizing therapies. Given that the mechanisms of apoptosis in HCC are not fully understood, our study offers new perspectives on the role of apoptosis in HCC metastasis and provides valuable biological clues for future research.

## Methods

### Data Collection and Processing

RNA-Seq data from TCGA-LIHC were downloaded using the TCGA GDC API, while the ICGC-LIRI-JP dataset was obtained from the HCCDB database. Gene expression data and related clinical information for liver cancer patients in GSE43619 were downloaded from the GEO database. The scRNA-seq data from GSE149614, containing 10 primary liver cancer samples and 8 adjacent non-tumor liver samples, were also downloaded from the GEO database.

The bulk RNA-Seq dataset was preprocessed as follows: (1) samples without clinical follow-up information were removed; (2) samples without survival status were excluded; (3) Ensemble IDs were converted to gene symbols; and (4) expression values for multiple gene symbols were averaged. After screening, a total of 370 primary tumor samples and 50 adjacent non-tumor control samples were retained in TCGA-LIHC. 212 hepatocellular carcinoma samples were retained from the ICGC-LIRI-JP dataset, and 88 tumor samples were kept from GSE43619.

### Single-Cell Transcriptome Clustering and Identification of DE-Anoikis Genes

For the scRNA-seq dataset, we applied filtering thresholds such that each gene had to be expressed in at least 5 cells, and each cell had to express at least 200 genes. Cells were retained if the proportion of mitochondrial genes was < 15% and the number of genes ranged between 50 and 6000. Data were then normalized using the SC Transform function, and principal component analysis (PCA) was performed using the RunPCA function. Batch effects were removed using the harmony package. The first 30 principal components were used for t-SNE dimensionality reduction. Cell subpopulations were clustered using the FindNeighbors and FindClusters functions (resolution = 0.1), and cell types were annotated based on marker genes from the CellMarker2.0 database.

The keyword "Anoikis" was searched on the GeneCards website (https://www.genecards.org/), and 211 genes with a score ≥ 1.5 were identified. To analyze the role of anoikis in hepatocellular carcinoma, we intersected 615 differentially expressed markers (logFC. threshold > 0.25) with the 211 anoikis-related genes. A total of 17 DE-Anoikis genes were identified.

### HCC Consistency Clustering and Functional Enrichment

Based on the DE-Anoikis genes, unsupervised clustering was performed on the TCGA-LIHC dataset using the R package "ConsensusClusterPlus" (version 1.58.0) 17. Kaplan-Meier (KM) survival curves for different subgroups and clusters were analyzed and plotted using the R packages "survival" (version 3.2.13) and "survminer" (version 0.4.9). Gene expression in different subgroups and clusters was visualized using the R package "ComplexHeatmap" (version 2.10.0).

### Modeling of Prognostic Risk Associated with Anoikis Resistance

To identify genes differentially expressed between C1 and C2 subtypes, we used the limma package (FDR < 0.05 and |log2FC| > log2(2)). Univariate Cox regression analysis of these genes was then performed using the "coxph" function to identify genes with prognostic significance. To further refine key genes, we applied the Least Absolute Shrinkage and Selection Operator (LASSO) algorithm using the R package "glmnet" (version 4.1.3) 18. This approach helped construct a penalty function, providing a more accurate model to address the issue of multicollinearity in regression analysis 19. Stepwise regression was performed using the Akaike information criterion (AIC) in the MASS package (version 7.3.54), starting with the most complex model and iteratively removing variables to reduce AIC. A smaller AIC value indicates a better model, suggesting a sufficient fit with fewer parameters.

The "coxph" function was used to perform multivariate Cox analysis of the hub genes, and the coefficients for each gene were determined. The risk score for each sample was calculated as the sum of the product of each gene and its coefficient. TCGA-LIHC samples were then categorized into high-risk and low-risk groups based on the optimal risk score cutoff points, which were identified using the "surv_cutpoint" function. Receiver operating characteristic (ROC) curve analyses were performed using the R package "timeROC" and nomograms were constructed using the "rms" package (version 6.2.0).

### Correlation Analysis Between Risk Score and Drug Sensitivity

The R package "oncoPredict" was used to predict drug IC50 values for the TCGA-LIHC dataset samples. Pearson correlation analysis was then conducted to evaluate the relationship between drug sensitivity and risk score, with p < 0.05 and |cor| > 0.5 considered statistically significant.

### Immunohistochemistry and Quantification of Hub Protein Expression in HCC

To validate the protein-level expression of the key protein G6PD in hepatocellular carcinoma (HCC) tissues, immunohistochemistry (IHC) was performed on formalin-fixed, paraffin-embedded (FFPE) tumor sections. The anti-G6PD primary antibody was purchased from Proteintech. Tissue preparation followed standard protocols established by the institutional pathology laboratory. Tissue samples were fixed in 4% paraformaldehyde, dehydrated, embedded in paraffin, sectioned, stained, and sealed according to the standard operating procedures (SOP) of the core facility. Quality control was ensured before imaging. Image analysis was conducted using Visiopharm (Microvis, Dangéuil) software. The “HDAB-DAB” color deconvolution protocol was applied for automated detection of the DAB-positive signal across whole-slide images. Area Density (Integrated Optical Density/Area) was computed, reflecting both the extent and intensity of G6PD-positive staining. This metric is independent of tissue area and thus provides a robust quantification. All regions of interest (ROIs) were manually defined to include the full tissue area. Positive staining thresholds were calibrated and standardized across all samples using a fixed color segmentation model to ensure consistency in quantification. After image analysis, area density and average optical density values were extracted and further visualized using R for statistical comparison between groups.

## Results

### Identification of DE-Anoikis

**Figure 1:**
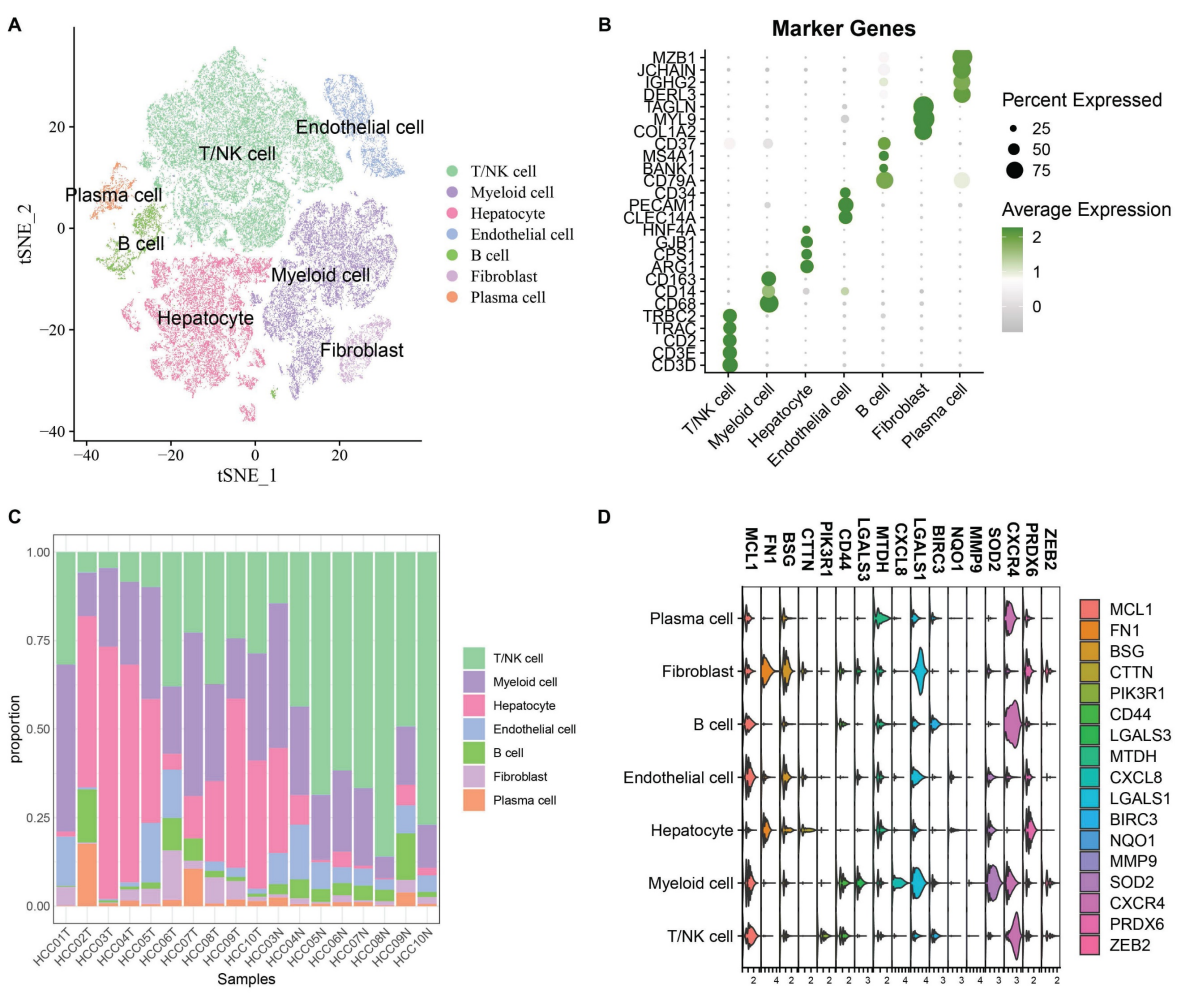
(A) Annotated t-SNE plot showing cell subpopulations. (B) Bubble plot of classical marker gene expression in different cell subpopulations. (C) Percentage of cell subpopulations in each sample. (D) Violin plots showing the expression levels of 17 DE-Anoikis genes in each cell type.

After cell filtering and normalization, subcluster analysis of the single-cell transcriptomes in GSE149614 was performed and visualized using the t-distributed stochastic neighbor embedding (t-SNE) approach. Quality Control of Single Cell Sequencing Data was showed in Supplementary Figure 1A total of 60,496 cells were retained and divided into 10 cell clusters. Based on the expression of classical marker genes, these clusters were annotated as T/NK cells, Myeloid cells, Endothelial cells, Fibroblasts, B cells, Hepatocytes, and Plasma cells (Fig. 1A-B). The proportions of various cell types in different samples are shown in Fig. 1C. To analyze the role of anoikis in hepatocellular carcinoma, 615 differentially expressed cell markers (logfc.threshold > 0.25) were intersected with 211 anoikis-related genes. A total of 17 DE-Anoikis genes were identified. The expression patterns of these 17 DE-Anoikis in individual cells are shown in Fig. 1D. MCL1, LGALS1, MTDH, and CXCR4 were widely distributed among various cell clusters, while SOD2 was predominantly expressed in myeloid cells, and FN1 was highly expressed in fibroblasts. These genes may act synergistically among different cell types to promote hepatocarcinogenesis.

### Consistent Clustering of HCC Based on DE-Anoikis with Prognostic Significance

**Figure 2:**
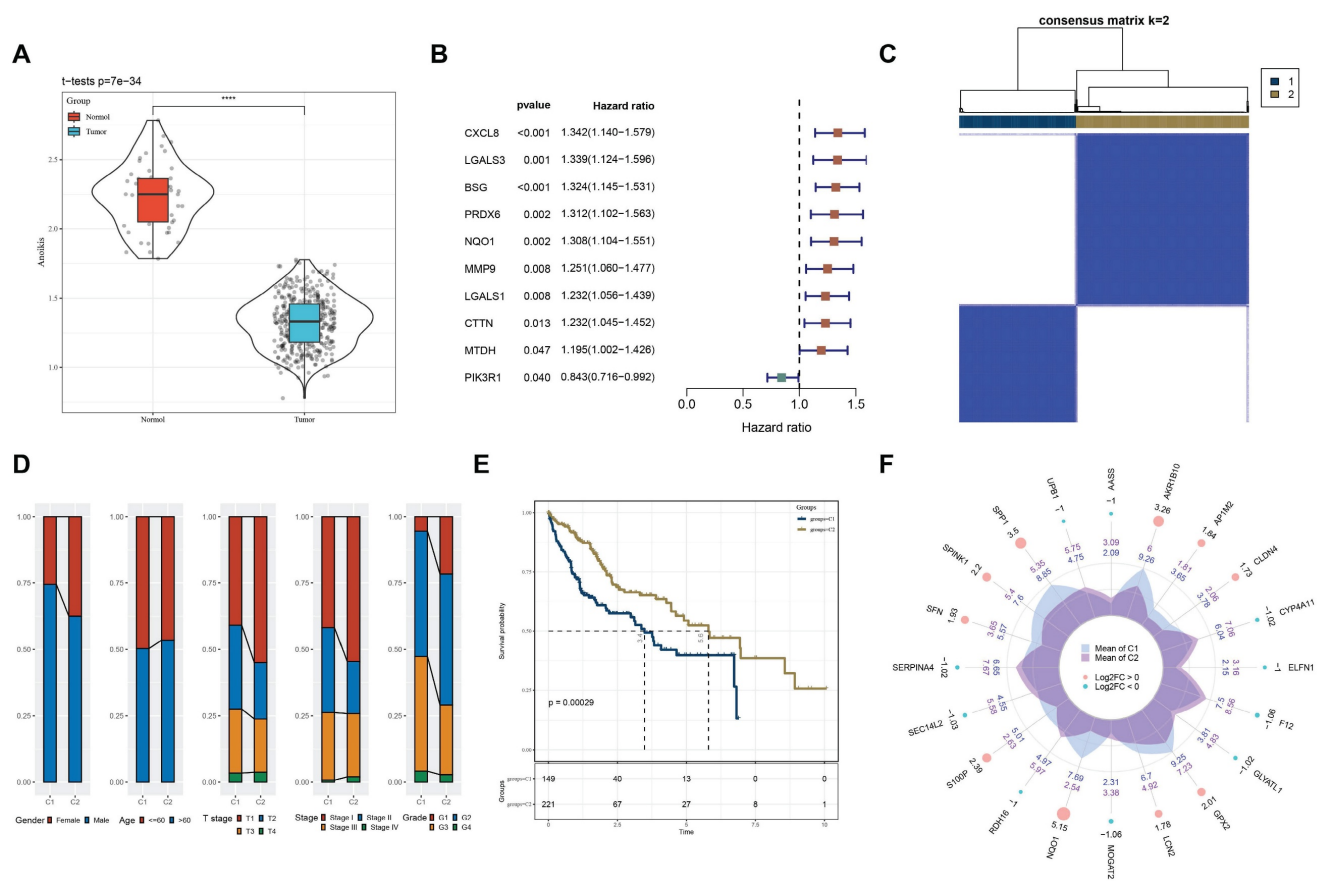
(A) Boxplot showing ssGSEA results, indicating significantly lower DE-Anoikis scores in cancer tissues compared to normal tissues. (B) One-way Cox analysis of DE-Anoikis in the TCGA-LIHC dataset, identifying 10 genes with p < 0.05. (C) Consensus clustering heatmap of the TCGA-LIHC dataset. (D) Clinicopathological characteristics of the two molecular subtypes in the TCGA-LIHC dataset. (E) Kaplan-Meier (KM) curves illustrating overall survival (OS) prognosis of the two molecular subtypes. (F) Radar plot showing the top 10 differentially expressed genes between C1 and C2 subtypes, where red dots represent upregulated genes and green dots represent downregulated genes.

We explored differences in anoikis resistance features between tumor and adjacent non-tumor tissues using the ssGSEA algorithm. DE-Anoikis-based enrichment scores indicated that tumor tissues had significantly lower DE-Anoikis scores compared to normal tissues, suggesting downregulation of anoikis regulation in tumor tissues (Fig. 2A). We subsequently analyzed the gene expression of DE-Anoikis in relation to prognosis and identified 10 DE-Anoikis genes significantly correlated with prognosis (p < 0.05), namely, CXCL8, LGALS3, BSG, PRDX6, NQO1, MMP9, LGALS1, CTTN, MTDH, and PIK3R1 (Fig. 2B).

Based on these 10 DE-Anoikis genes, HCC patients in the TCGA-LIHC dataset were subjected to molecular subtyping. The two optimal clusters identified were named C1 and C2 (Fig. 2C, Supplementary Table 1).

To explore the impact of anoikis resistance on the prognostic characteristics of HCC patients, clinicopathological features were compared between the C1 and C2 subtypes. The C1 subtype had higher clinical stage and grade (Fig. 2D). Kaplan-Meier (KM) analysis showed that patients in the C1 subtype had significantly worse overall survival compared to the C2 subtype (p < 0.05, Fig. 2E). Differentially expressed genes between the clusters were identified (FDR < 0.05 and |log2FC| > log2(2), Supplementary Table 2), and the top 10 up- and down-regulated genes were visualized using radar plots (Fig. 2F). To further verify the tissue origin of the differentially expressed genes (DEGs) between the C1 and C2 subtypes, we performed enrichment analysis at the single-cell level using the AUCell algorithm. The enrichment scores of the DEG set were predominantly distributed in hepatocyte populations in the single-cell RNA-seq dataset GSE149614, indicating that these genes are mainly derived from resident liver cells within the tumor tissue rather than detached cells (Supplementary Fig. 2). Furthermore, KEGG enrichment analysis revealed that these DEGs were significantly enriched in pathways such as the PPAR signaling pathway, Glycolysis/Gluconeogenesis, Carbon metabolism, and IL-17 signaling pathway, all of which are closely associated with liver cell function and metabolic reprogramming. These findings strongly support the notion that the DEGs originate from functional hepatocytes within tumor tissues.

**Figure 3:**
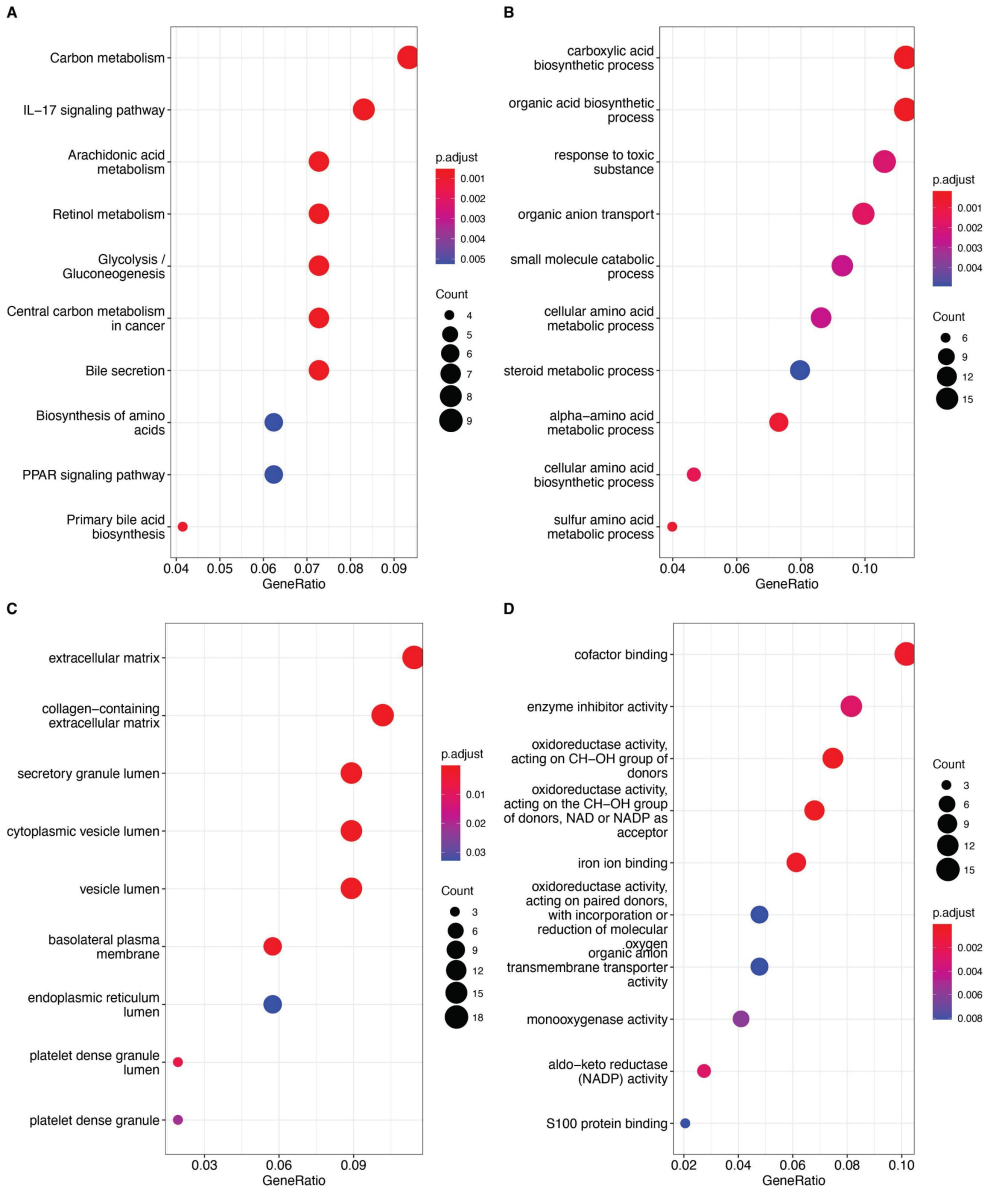
A: Bubble plot of KEGG pathway enrichment analysis, with bubble size representing the number of genes and color representing significance (p-value). B: Bubble plot of GO Biological Process (GO_BP) enrichment analysis. C: Bubble plot of GO Cellular Component (GO_CC) enrichment analysis. D: Bubble plot of GO Molecular Function (GO_MF) enrichment analysis. The significance of each entry is represented by the color, increasing sequentially from blue to red.

We then used 161 differentially expressed genes from the HCC subtypes for functional enrichment analysis to clarify differential biological functions and pathways. Interestingly, many metabolism-related pathways, such as carbon metabolism, IL-17 signaling pathway, glycolysis, and amino acid metabolism, were significantly enriched (Fig. 3A-D). These findings suggest that differential regulation of anoikis resistance may be linked to metabolic reprogramming in tumor tissues.

### Construction and Validation of a Clinical Prognostic Model

**Figure 4:**
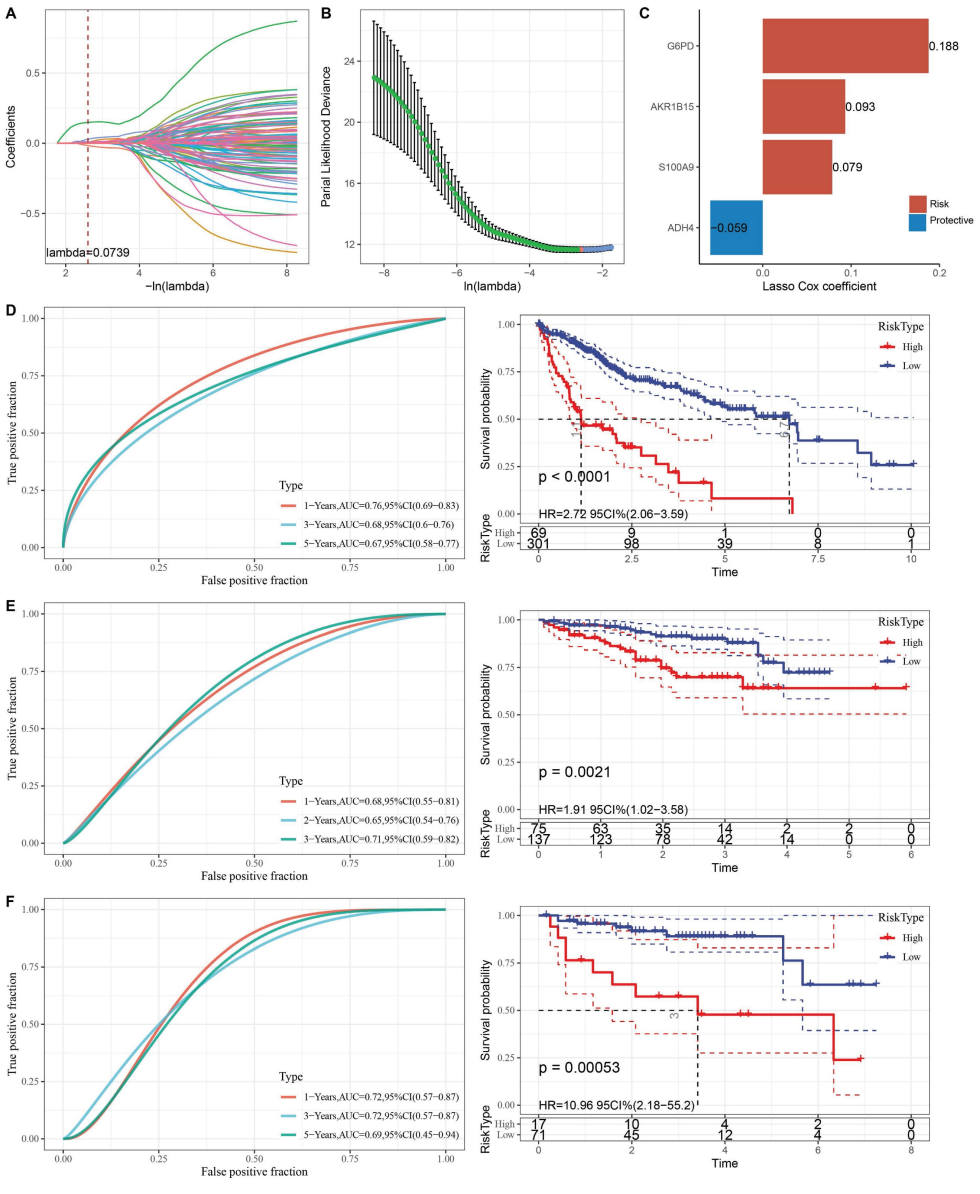
(A) Trajectory of each independent variable with different lambda values during LASSO regression. (B) Confidence intervals under each lambda value. (C) Prognostic model constructed based on selected genes. (D) Clinical prognostic model and KM survival curves for the TCGA-LIHC dataset, shown from left to right. (E) Validation of the clinical prognostic model and KM survival curves for the ICGC-LIRI-JP dataset, shown from left to right. (F) Validation of the clinical prognostic model and KM survival curves for the GSE43619 dataset, shown from left to right.

To further clarify the prognostic significance of anoikis resistance in HCC, we constructed a clinical prognostic model based on differentially expressed genes (DEGs). First, 161 DEGs were analyzed using univariate Cox regression, and 111 genes with significant prognostic value were identified (p < 0.05). LASSO regression was then used to reduce the number of prognostic genes for risk modeling. The trajectory of lambda for each independent variable is shown in Fig. 4A-B. Ten-fold cross-validation was performed, and confidence intervals were analyzed for each lambda. The model reached its optimum at lambda = 0.0739, and six genes were selected. We then used stepwise multivariate regression analysis to further refine the model, resulting in the identification of four influential prognostic genes and their coefficients (Fig. 4C). The RiskScore for each sample was calculated using the following formula: RiskScore = 0.188 * G6PD + 0.093 * AKR1B15 + 0.079 * S100A9 - 0.059 * ADH4, where G6PD, AKR1B15, and S100A9 are risk factors, and ADH4 is a protective factor.

Based on the optimal cutoff point, LIHC patients were categorized into high-risk and low-risk groups. The classification efficiency for prognostic prediction at one, three, and five years was analyzed, with the RiskScore reaching 0.76, 0.68, and 0.67, respectively, for survival prediction (Fig. 4D). KM analysis showed that the overall survival rate of high-RiskScore patients was significantly lower than that of low-RiskScore patients. The clinical prognostic model was validated using the ICGC-LIRI-JP (Fig. 4E) and GSE43619 (Fig. 4F) validation sets, where similar results were observed as in the training set.

**Figure 5:**
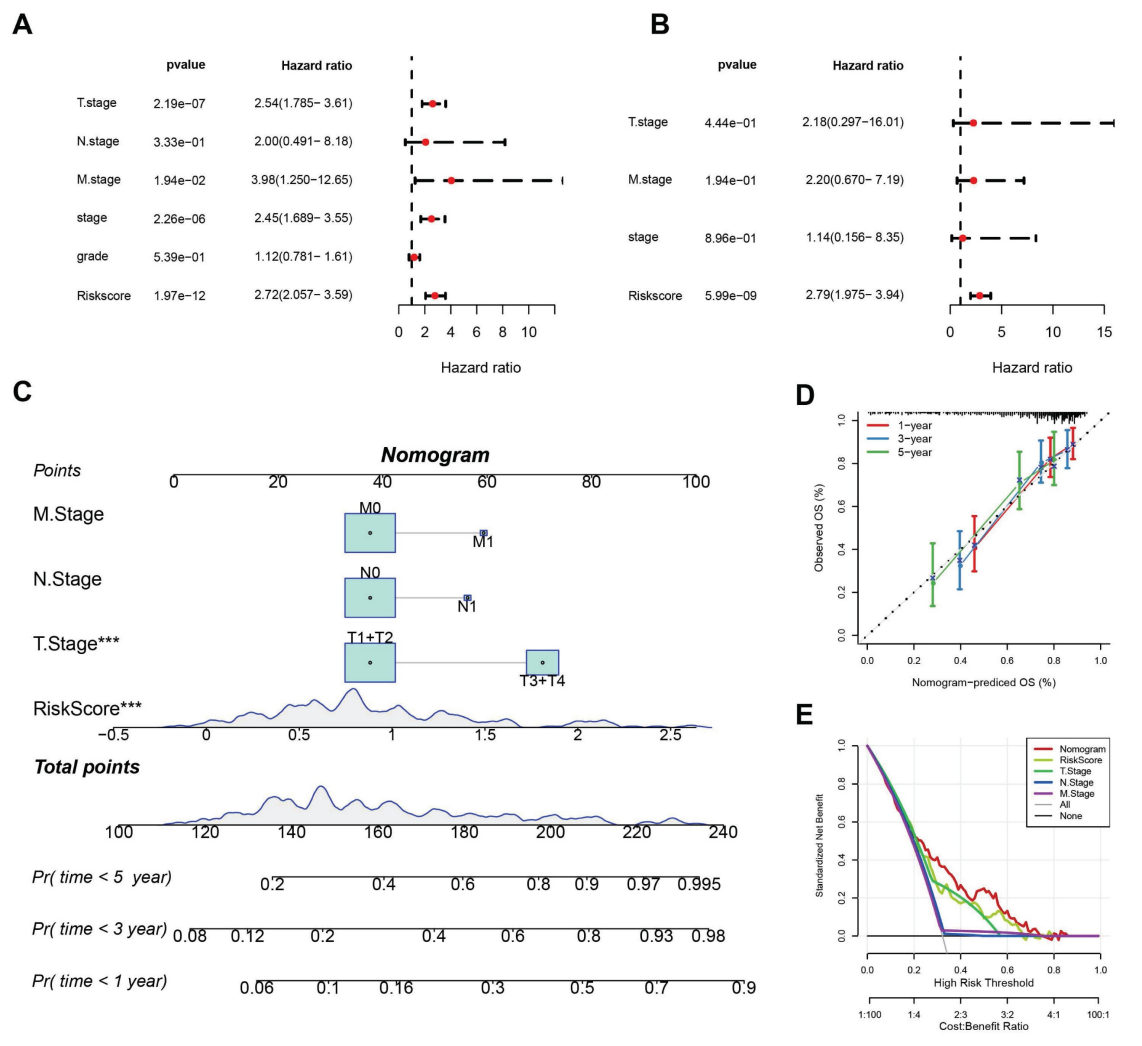
(A) Results of univariate Cox analysis. (B) Results of multivariate Cox analysis. (C) Column plot combining RiskScore with clinical characteristics. (D) Calibration curves for the column plot. (E) Decision curve analysis (DCA) for the column plot.

To determine whether RiskScore was independent of other clinical factors, univariate and multivariate Cox regression analyses were performed in the TCGA-LIHC dataset (Fig. 5A-B). Univariate analyses showed that T stage, M stage, overall stage, and RiskScore were all significant prognostic factors. Multivariate analysis confirmed that RiskScore was an independent risk factor for HCC prognosis, with the highest hazard ratio (HR = 2.79).

To quantify the risk assessment and survival probability of patients in the TCGA-LIHC dataset, a nomogram combining T stage, N stage, M stage, and RiskScore was created (Fig. 5C). The nomogram indicated that RiskScore had the greatest impact on survival prediction. Calibration curves showed good predictive accuracy for one-, three-, and five-year survival, as they closely aligned with standard curves (Fig. 5D). Decision curve analysis (DCA) indicated that the nomogram offered significant benefits compared to extreme curves, demonstrating strong predictive ability compared to other individual clinical features (Fig. 5E).

### Evaluation and IHC validation of classical apoptotic resistance pathway

**Figure 6:**
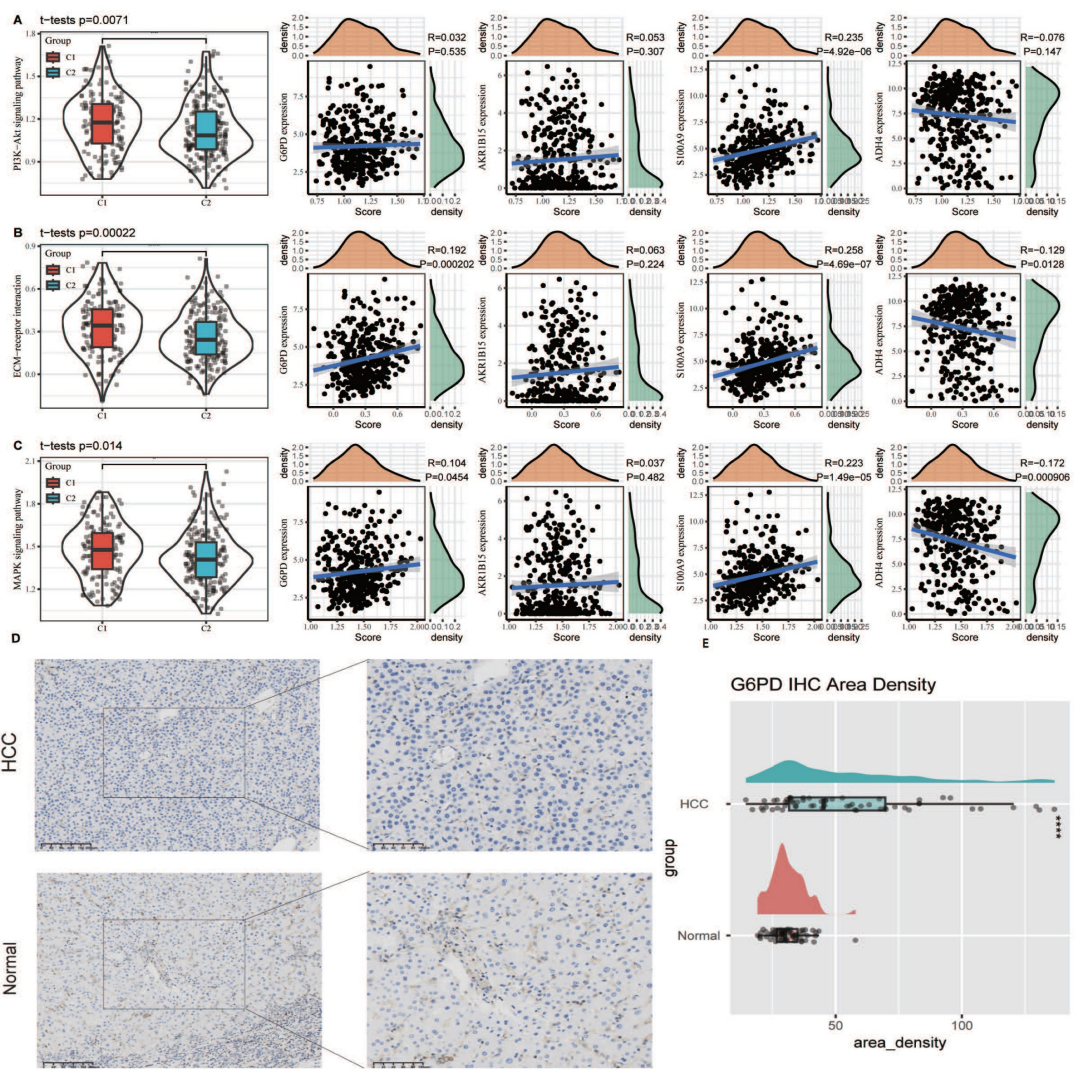
Association of anoikis resistance pathways with model genes and validation of G6PD expression via immunohistochemistry. (A-C) ssGSEA was used to assess the activity of canonical anoikis resistance-related pathways, including the PI3K-Akt signaling pathway, ECM-receptor interaction, and MAPK signaling pathway, based on KEGG gene sets. (D) Immunohistochemical staining of G6PD in paired HCC and adjacent non-tumor tissues demonstrated markedly higher expression of G6PD in tumor tissues. (E) Quantitative analysis of the Area Density from IHC staining confirmed that G6PD protein levels were significantly upregulated in HCC tissues compared to adjacent non-tumor controls (p < 0.05).

To elucidate the relationship between classical apoptosis and anoikis resistance, we examined the expression levels of key apoptosis markers (e.g., BAX, BCL2, CASP3) between tumor and normal tissues, as well as between the C1 and C2 subtypes. Significant differential expression was observed (Supplementary Fig. 3). Additionally, we analyzed the activity of key signaling pathways involved in anoikis resistance, including the PI3K-Akt signaling pathway, ECM-receptor interaction, and MAPK signaling pathway, as defined in the KEGG database. Single-sample gene set enrichment analysis (ssGSEA) revealed that these pathways were significantly more active in the C1 subtype (p < 0.05). Correlation analysis showed that the expression of G6PD and S100A9 was positively associated with pathway activity scores, while ADH4 expression was negatively associated. These findings suggest that the biomarkers defined in our model are closely linked to canonical anoikis resistance mechanisms and may contribute to apoptotic dysregulation in HCC (Fig. 6A-C). Furthermore, we conducted IHC on the G6PD risk factor in HCC and adjacent tissues. The results showed that the expression of G6PD was significantly increased in HCC tissues compared to adjacent tissues (Fig. 6D). The Area Density of G6PD staining for all pathological sections also showed significant high expression in HCC tissues (Fig. 6E).

### Sensitivity analysis of chemotherapy drugs based on risk score

**Figure 7:**
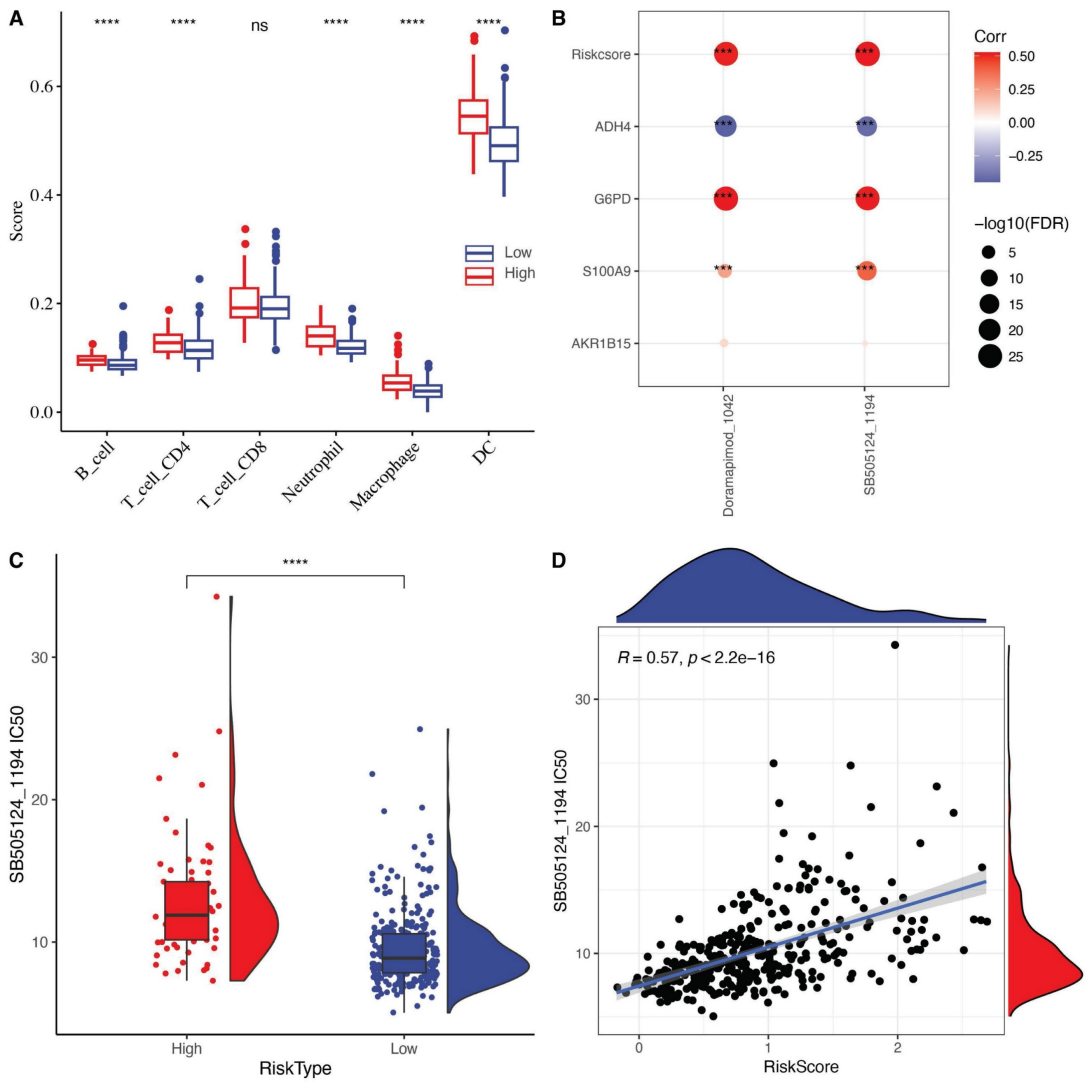
(A) Differences in immune cell scores between high and low-risk groups in the TCGA-LIHC cohort. (B) Correlation analysis between RiskScore in the TCGA-LIHC dataset and the expression of key genes in the model and drug IC50. (C) Comparison of IC50 values of SB505124_1194 drug between high and low-risk groups. (D) The relationship between the IC50 and risk score of SB505124_1194 drug relevance.

To elucidate the relationship between RiskScore and the immune microenvironment, we used the TIMER software to assess immune cell profiles. The results showed significant differences in immune cell infiltration between high-risk and low-risk groups (Fig. 7A). Unexpectedly, immune cell infiltration was significantly higher in the high-risk group compared to the low-risk group. We speculate that the acquisition of anoikis resistance makes tumor cells more likely to detach from the extracellular matrix, which elicits a response from the immune surveillance system. Although immune cell infiltration may enhance the anti-tumor response in the tumor microenvironment (TME), the highly metastatic nature of this tumor type still results in a poor prognosis.

We further explored the relationship between RiskScore and drug sensitivity by calculating the half-maximal inhibitory concentration (IC50) values for each drug in the TCGA-LIHC samples. We identified a significant correlation between two drugs and RiskScore (FDR < 0.05 and |cor| > 0.5). RiskScore, ADH4, G6PD, and S100A9 were significantly correlated with the IC50 values of SB505124_1194 and Doramapimod_1024 (Fig. 7B). Among these, the IC50 value of SB505124_1194 was higher in the high-RiskScore group, indicating that patients with a high RiskScore were resistant to this drug, while those in the low-risk group were more sensitive (Fig. 7C-D). Among the screened compounds using the oncoPredict algorithm, SB505124_1194 (corresponding to SB505124) exhibited a strong inverse correlation with the anoikis-related RiskScore, indicating that patients in the low-risk group may be more responsive to this compound. SB505124 is a selective inhibitor of the TGF-β type I receptors ALK4/5/7, which blocks the phosphorylation of Smad2/3 and downstream TGF-β signaling. In advanced hepatocellular carcinoma, TGF-β signaling contributes to epithelial-mesenchymal transition (EMT), metastasis, and anoikis resistance. By inhibiting this pathway, SB505124 may enhance sensitivity to anoikis and promote apoptosis in tumor cells, aligning with the core mechanisms explored in this study. Although SB505124 is currently in preclinical development and not yet in clinical use, the integration of its sensitivity profile with our prognostic model suggests potential therapeutic value for low-risk HCC patients and highlights avenues for personalized treatment strategies.

## Discussion

It has been shown that when cancer cells detach from the extracellular matrix (ECM), they must overcome several obstacles to survive 10, 20. One critical step in this process is acquiring resistance to anoikis 21, 22. Different pathways drive anoikis, ultimately converging on cysteine asparaginase activation, nucleic acid endonuclease activation, and DNA fragmentation, leading to cell death 23, 24. This process can be triggered by mitochondrial perturbation or cell surface death receptors 25. ECM detachment sends signals to induce cell death through multiple mechanisms 26. Resistance to anoikis occurs via various mechanisms, including changes in integrins, overexpression or mutation of growth factor receptors, ECM stiffness, production of pro-survival soluble factors, epithelial-mesenchymal transition (EMT), and metabolic dysregulation 27. Although many studies have preliminarily clarified the role of anoikis in various cancers, including HCC, new perspectives are needed to better understand the comprehensive prognostic role of anoikis in HCC, given the wide range of pathways involved and its critical role in tumor metastasis.

In this study, using a single-cell dataset and multiple HCC transcriptome cohorts, we established the significant prognostic value of anoikis resistance in HCC. We identified molecular subtypes of HCC based on anoikis features, revealing clear prognostic and functional differences among the subtypes. Metabolic reprogramming of tumor cells appeared to be significantly correlated with the acquisition of anoikis resistance. Huakan Zhao et al. demonstrated that STIM1 stabilized the Snail1 protein during tumor growth by activating the CaMKII/AKT/GSK-3β pathway. Subsequently, upregulated Snail1 inhibited STIM1/SOE during metastasis, while STIM1 repair significantly reduced Snail1-induced anoikis resistance and metastasis. Thus, STIM1 plays a role in coordinating invasion and metastasis by reprogramming HCC metabolism 28. Updated studies have shown that ECM isolation promotes the accumulation of single-carbon metabolites, induces anoikis regulatory pathway genes, and increases total DNA methylation 29. This is consistent with our findings. Our results also indicated that sugar metabolism and amino acid metabolism, along with carbon metabolism, are involved in anoikis resistance acquisition, suggesting a complex metabolic network that still requires further investigation.

Based on differentially expressed genes associated with anoikis resistance and prognostic significance, we identified key hub genes for this process: G6PD, AKR1B15, S100A9, and ADH4. It has been shown that G6PD expression is regulated by the GLV9-AMPK axis, leading to glycolipid metabolism reprogramming, disrupting redox homeostasis, and inducing anoikis 30. The roles of AKR1B15, S100A9, and ADH4 in anoikis are less established. Notably, ADH4 expression and its regulation of NAD+, NAD+/NADH ratio, and ATP concentrations are important in redox homeostasis in HCC, potentially contributing to anoikis resistance in HCC 31. Our clinical prognostic model, constructed based on anoikis-related hub genes, demonstrated good prognostic efficacy in multiple validation sets, corroborating the role of anoikis resistance in HCC progression. These results provide further insight into the role of anoikis in HCC. However, more studies are needed to clarify the comprehensive anoikis regulatory network in HCC and to determine whether this biological signature could be a potential therapeutic target for advanced HCC.

## Conclusion

This study characterized the gene panel of DE-Anoikis and defined anoikis resistance-associated HCC molecular subtypes, revealing significant metabolic differences across anoikis resistance traits. We identified four anoikis-associated signatures using LASSO and constructed a clinical prognostic model with good efficacy. The model also identified two drugs associated with risk scores, which could contribute to personalized cancer medicine.

## Supplementary Material

Supplementary figures and tables.
