# Supplementary material for: Exploration of the relationship between apoptosis related characteristic genes and the prognosis of HCC
Source: J Cancer. 2025 Jul 28;16(11):3485–96. doi: 10.7150/jca.114359 (PMC12374940; doi:10.7150/jca.114359)

Supplementary Table 1 Sample Information of HCC Subtypes C1 and C2;

Supplementary Table 2 Differentially Expressed Genes between HCC Subtypes C1 and C2.

Supplementary Figure 1 Quality Control of Single Cell Sequencing Data

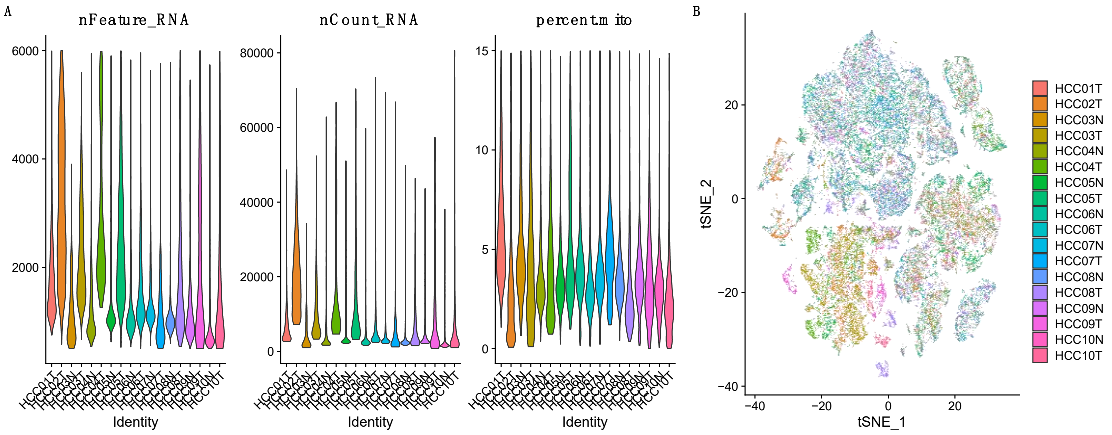

Supplementary Figure 2 Identifying the tissue sources of differentially expressed genes based on AUCell method

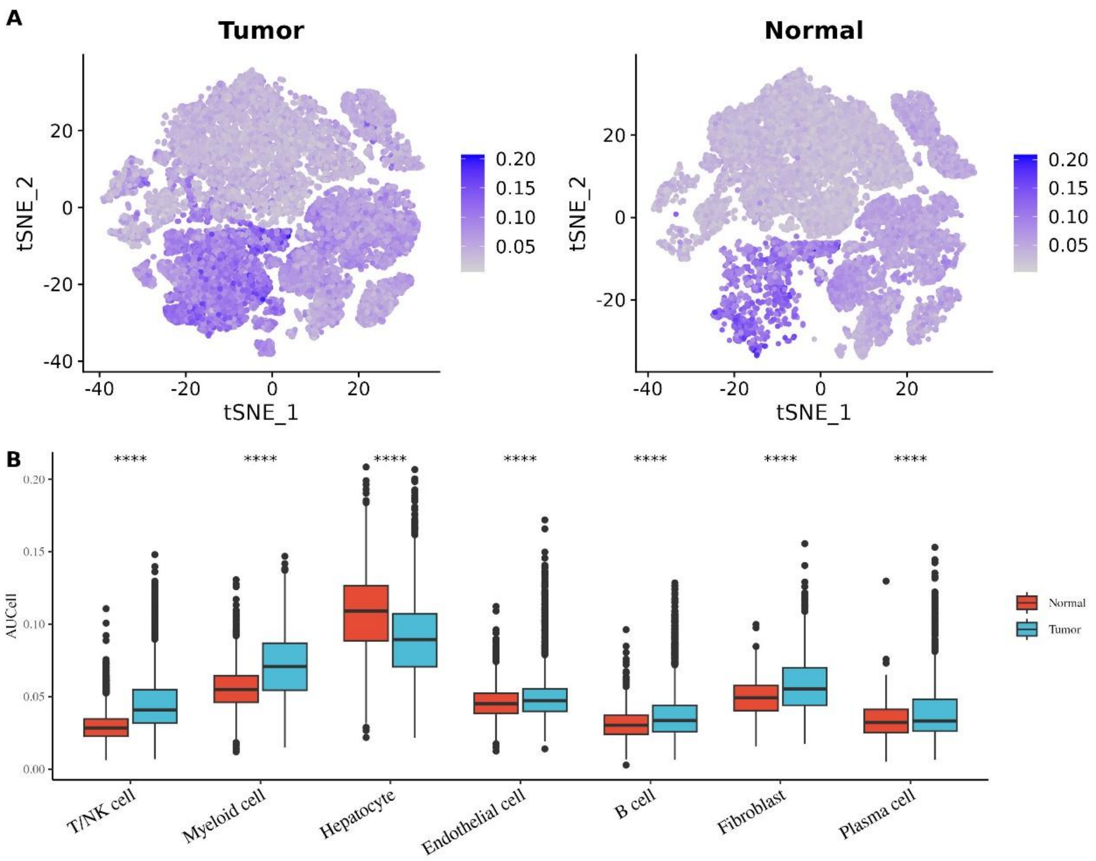

Supplementary Figure 3 Differential expression of classic apoptosis markers between normal and tumor tissues, as well as C1 and C2 subtypes

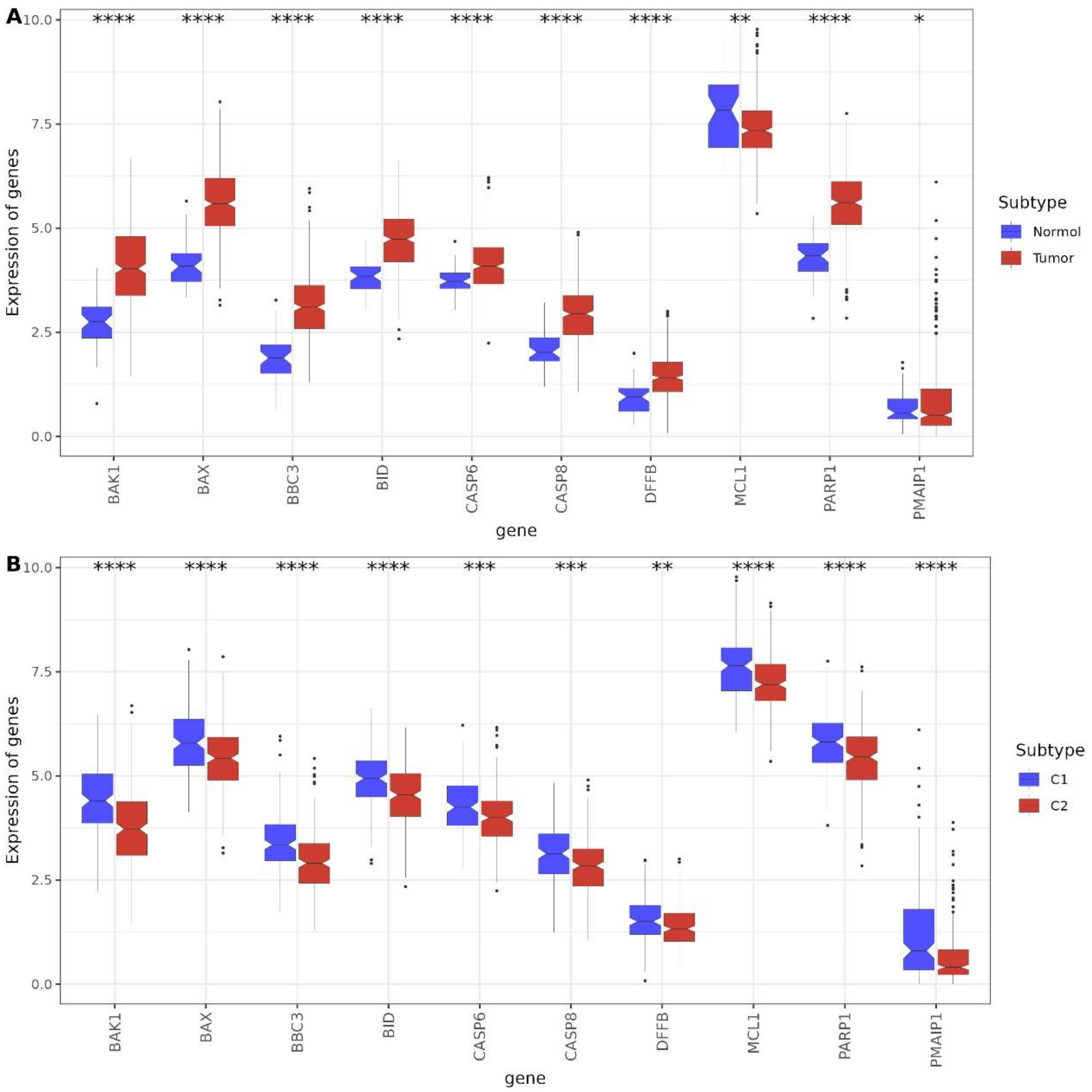

Supplement: Supplementary file 1 — Supplementary figures and tables. [file jcav16p3485s1.pdf]
